# African swine fever in Nepal: risk factors, impacts, and strategies for control

**DOI:** 10.1016/j.soh.2026.100150

**Published:** 2026-02-12

**Authors:** Sameer Thakur, Kshitiz Shrestha, Ram Chandra Acharya, Parikshya Gurung, Surendra Karki

**Affiliations:** a Animal Service Department, Dhangadhi Sub-Metropolitan City Office, Dhangadhi 10900, Nepal; b Melbourne Veterinary School, The University of Melbourne, Melbourne, Victoria 3010, Australia; c National Vaccine Production Laboratory, Department of Livestock Services, Kathmandu 44600, Nepal; d Department of Livestock Services, Ministry of Agriculture and Livestock Development, Lalitpur 44700, Nepal; e Food and Agriculture Organization of the UN (FAO), Emergency Centre for Transboundary Animal Diseases (ECTAD), Kathmandu 44600, Nepal

**Keywords:** African swine fever, Pig farming, Epidemiology, Socio-economic impact, Transboundary animal diseases, Risk factor, Nepal

## Abstract

African swine fever (ASF) has emerged as a major threat to Nepal’s pig farming sector since its first outbreak in March 2022. The disease has caused significant declines in both the pig population and pork production, severely affecting rural communities reliant on pig farming for their livelihoods. ASF’s spread is facilitated by key risk factors, including swill feeding, informal cross-border trade, and poor on-farm biosecurity measures. Additionally, seasonal trends, particularly during the monsoon, exacerbate the spread of the disease, especially in areas with high pig density. Control efforts face numerous challenges, including limited veterinary infrastructure, inadequate surveillance systems, gaps in legislation, and a lack of awareness among farmers about biosecurity practices. This study presents a comprehensive analysis of ASF’s epidemiology in Nepal, exploring the disease’s socio-economic impact and identifying key control challenges. The paper provides targeted recommendations to improve veterinary services, strengthen surveillance systems, enforce biosecurity measures, and enhance cross-border cooperation. It also emphasizes the importance of fostering community engagement and developing effective policy frameworks to ensure long-term ASF prevention and control, ensuring a more resilient pig farming sector in Nepal.

## Graphical abstract

**Figure:**
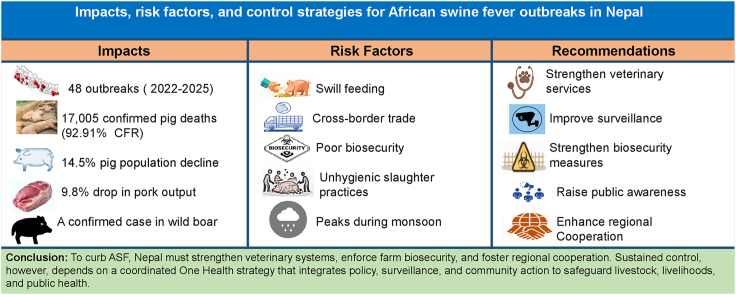

## 1 Introduction

African swine fever (ASF) is a contagious viral disease affecting domestic and wild pigs, characterized by high mortality and significant socio-economic losses among pig farmers [1,2]. The disease is caused by the African swine fever virus (ASFV), a large, enveloped DNA virus belonging to the genus *Asfivirus*, family *Asfarviridae* [3]. ASFV is primarily transmitted through direct contact with infected pigs, contaminated pork products, swill feeding, and infected bodily fluids; indirect transmission through fomites, contaminated vehicles and equipment; and vector-borne transmission through bites from soft ticks of the *Ornithodoros* genus [4,5]. ASFV strains vary in virulence, causing clinical forms ranging from peracute and acute to subacute and chronic. Peracute ASF, caused by highly virulent strains, progresses rapidly with high fever (up to 42 °C), loss of appetite, extreme fatigue, and, in some cases, sudden death with no prior symptoms [6]. Acute ASF, from moderately or highly virulent isolates, presents with high fever (40–42 °C), lethargy, anorexia, centripetal cyanosis of the ears, snout, limbs, and abdomen, frequent skin hemorrhages, blood-stained diarrhea, and respiratory distress, often leading to up to 100 % mortality within a week [6,7]. Subacute ASF, typically caused by moderately virulent strains, presents with similar but milder signs than acute ASF, including moderate to high fever, with mortality rates ranging from 30 % to 70 % [7]. Chronic ASF, caused by low virulent strains, progresses slowly, with clinical signs including skin necrosis, emaciation, occasional abortion, respiratory distress, and weight loss, but lower mortality. Chronic survivors can become long-term carriers, shedding the virus without clinical symptoms and contributing to ongoing transmission [6,7]. The virus primarily targets mononuclear phagocytic cells such as macrophages, disrupting immune responses and leading to extensive tissue damage [3,8]. Diagnosis relies on molecular and serological methods, primarily PCR and enzyme-linked immunosorbent assay (ELISA), respectively, along with virus isolation for confirmation [9]. However, control remains challenging due to the absence of effective vaccines and treatments [10].

ASF was first introduced to Asia in China in August 2018 and rapidly spread across the region, reaching Vietnam, Cambodia, the Philippines, and Republic of Korea by 2019 [11,12]. The expansion continued into South and Southeast Asia in subsequent years. ASF caused devastating losses across Asia, with China experiencing the most severe impact, losing over 100 million pigs and incurring an estimated economic cost of 111.2 billion U.S. dollar between 2018 and 2020 [13,14]. Other nations, including Vietnam, the Philippines, Republic of Korea, and India, also suffered substantial agricultural and economic damage due to widespread pig mortality and culling efforts [15,16,17,18,19,20,21,22,23]. These regional experiences highlight the serious impacts of ASF on livelihoods and economies, providing the critical context for understanding its emergence and impact in Nepal.

In Nepal, pig farming is still emerging, gradually shifting from traditional, small-scale methods to more semi-commercialized and commercialized systems. Ethnic groups such as Magar, Gurung, Tamang, Rai, Limbu, Tharu, Kami, and Damai have been traditionally and predominantly engaged in pig farming and consumption of pork in Nepal [24]. However, with rising urban demand and commercialization, driven by increased urbanization and cultural shifts, pig farming and pork consumption are now becoming increasingly common among all communities [25]. Pork currently accounts for 7.56 % of total meat production in Nepal, ranking fourth behind chicken, buffalo meat, and chevon (goat meat) [26]. As pig farming continues to grow and demand for pork rises, recent growth trends suggest that pork is likely to play an increasingly important role in Nepal’s national meat economy.

Despite the potential, the development of piggery sector in Nepal is challenged by several factors ranging from socio-cultural taboos, lack of effective marketing infrastructure, high feed costs, lack of good breeds and health problems such as the classical swine fever (CSF). The emergence of ASF in March 2022 has further compounded existing challenges in Nepal’s pig sector [27]. Given the importance of pig farming for livelihoods and food security in Nepal, ASF presents major challenges for disease control, economic stability, and the future development of this sector [28]. This paper explores the epidemiological patterns of ASF outbreaks in Nepal since its initial detection, assesses their impact, examines the control challenges, and discusses potential strategies for more effective prevention and containment. To the best of our knowledge, this is the first comprehensive study analyzing these aspects of ASF outbreaks in Nepal, uniquely integrating national surveillance data of ASF outbreaks, mapping epidemiological trends, and comparing outbreaks with the geographical distribution and production trends of pigs in Nepal.

## 2 Geographical distribution and production trends of pigs in Nepal

**Fig. 1:**
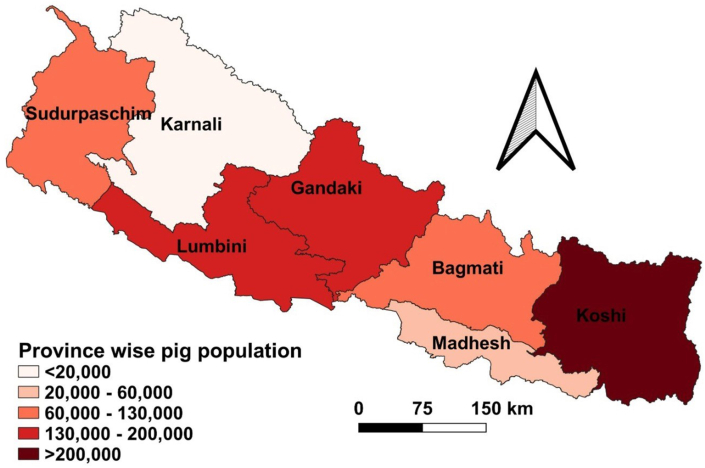
Province-wise distribution of pig population in Nepal during fiscal year 2022/23, represented using a color gradient from lowest to highest density (in thousands) (data sourced from Statistical Information on Nepalese Agriculture 2022/23 [26]; https://giwmscdnone.gov.np/media/pdf_upload/MOALD-Statical-Book-Magre-2081-Final_wgfs8ph.pdf; map created by the authors using QGIS software, version 3.22).

The domestic pig population in Nepal, as of Nepalese fiscal year 2022/23, is 1.357 million, accounting for 5.58 % of the country’s total livestock population [26]. Out of the seven provinces of Nepal, Koshi (57.51 %) has the highest pig population, followed by Lumbini (14.20 %) and Gandaki (9.85 %), while the lowest populations are found in Madhesh (1.64 %) and Karnali (1.17 %) provinces (Fig. 1) [26].

**Fig. 2:**
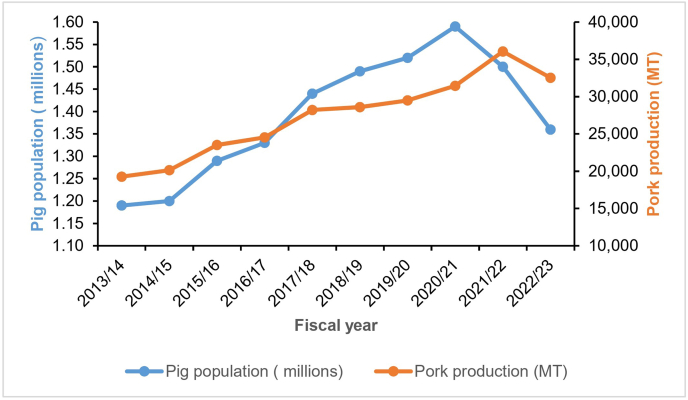
Trends in Nepal’s pig population (in millions) and pork production (in metric tons, MT) from fiscal year 2013/14 to 2022/23 (data sourced from Statistical Information on Nepalese Agriculture 2022/23) [26]; https://giwmscdnone.gov.np/media/pdf_upload/MOALD-Statical-Book-Magre-2081-Final_wgfs8ph.pdf).

An analysis of pig population trends over the past 10 years in Nepal, data from 2013/14 to 2020/21 show a steady increase of 33.50 %, rising from 1.190 million to a peak of 1.588 million, reflecting strong growth in pig farming. However, in the last two years, the population declined significantly by 14.50 % [26]. Similarly, pork production nearly doubled, increasing by 87.13 % from 19,269 metric tons (MT) in 2013/14 to 36,059 MT in 2021/22, but dropped sharply by 9.80 % to 32,533 MT in 2022/23 (Fig. 2) [26]. These declines coincide with the emergence and spread of ASF outbreaks across Nepal.

Pig farming in Nepal remains predominantly traditional, with about 73 % of pigs raised under such systems, which include both scavenging and backyard farming practices [29]. In traditional settings, pigs are allowed to forage freely during the day and are kept in small shelters at night, where they are fed agricultural by-products, and food scraps like vegetable peels and leftover cooked food. Indigenous pig breeds such as Hurrah, Chwanche, and Bampudke are primarily raised in this manner, particularly among ethnic communities in the Terai and hilly regions [30]. While this approach is cost-effective and more widely practiced, it poses higher risks of disease transmission, food contamination, and parasite infestations due to the lack of structured management practices. In contrast, a small proportion of pigs are raised under commercialized systems, including semi-intensive and intensive farming, typically found in the peri-urban areas. Under the semi-intensive system, pigs are usually confined but may have limited open areas for grazing, while in the intensive system, pigs are kept fully indoors and are exclusively fed commercial rations [24]. Exotic breeds such as Yorkshire, Landrace, Duroc, Hampshire, Meishan, and Tamworth are primarily raised under commercial farming systems [29]. While the commercial sector is expanding, it remains limited by high initial investment costs, infrastructure requirements, and the relatively slow adoption of fully commercial, intensive farming systems in the country.

## 3 Methodology for data collection, analysis and synthesis

**Fig. 3:**
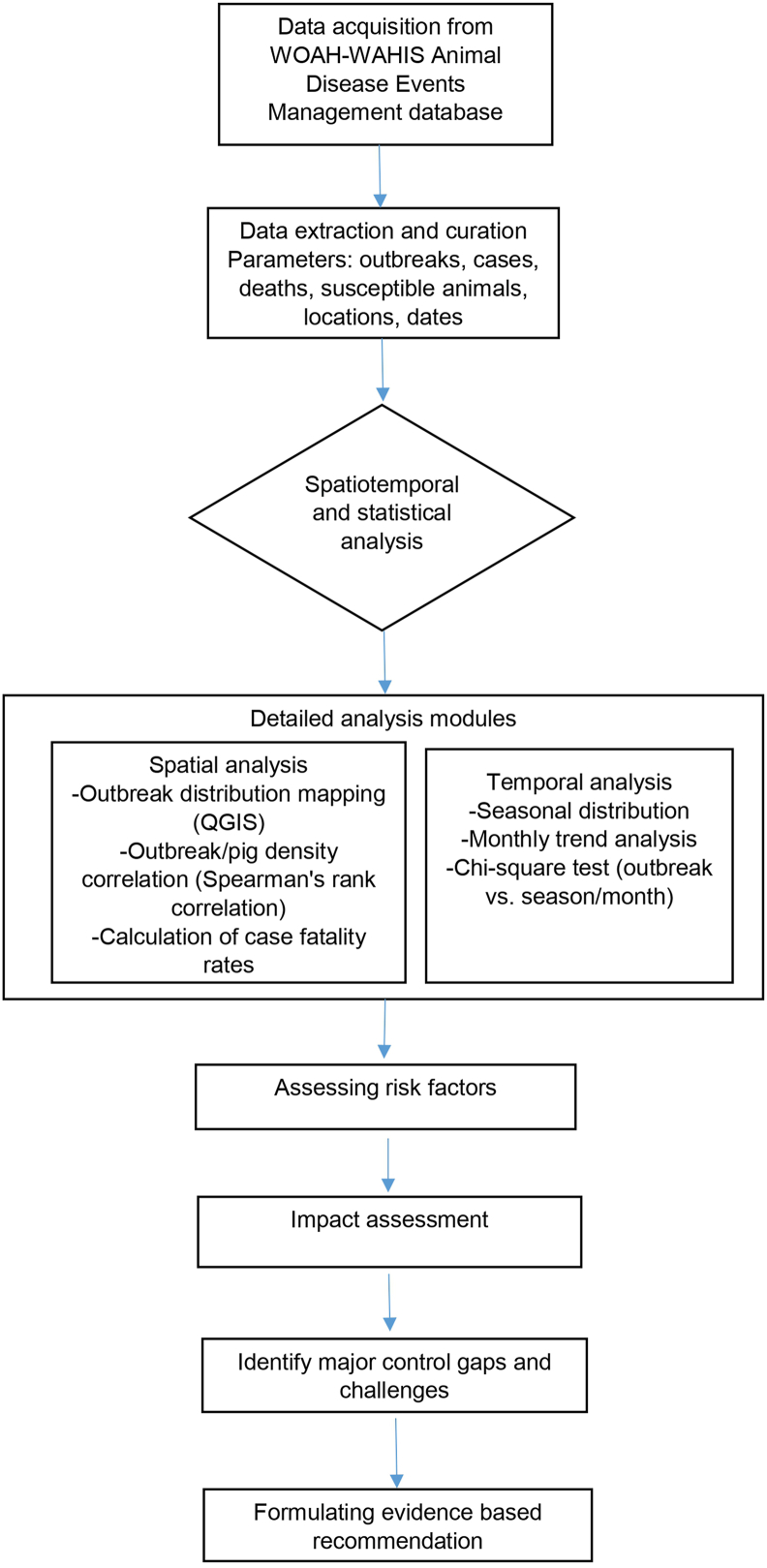
Flowchart of the methodology used to analyze ASF outbreaks in Nepal. Abbreviations: WOAH, World Organisation for Animal Health; WAHIS, World Animal Health Information System; QGIS, Quantum Geographic Information System.

This review employed a systematic approach to analyze the ASF situation in Nepal, from data collection to the formulation of control recommendations. Initially, outbreak data were gathered from the publicly available World Animal Health Information System (WAHIS) Animal Disease Events Management database, based on data submitted by Nepal’s designated veterinary authority to the World Organisation for Animal Health (WOAH) via WAHIS [31]. The collected data were then synthesized to generate epidemiological insights. These insights, combined with a review of existing literature and local context, were used to identify major control gaps and challenges, ultimately leading to the development of evidence-based recommendations for Nepal. The flowchart of methodology is shown in Fig. 3.

## 4 Epidemiology of ASF in Nepal

On 30 March 2022, the first confirmed ASF outbreak in Nepal was detected in domestic pigs aged 3–5 months in Kathmandu Valley (Bagmati Province). The disease was first confirmed through real-time PCR at the Central Veterinary Laboratory (CVL) in Kathmandu. Further confirmation and characterization were conducted by the Australian Animal Health Laboratory (AAHL), currently known as the Australian Centre for Disease Preparedness (ACDP), in Geelong, where they confirmed it to be Genotype Ⅱ, the strain responsible for recent Eurasian outbreaks, which is known for high virulence and rapid spread. This led to the notification of the first ASF case in Nepal to the WOAH in May 2022 [27].

Since then, a total of 48 ASF outbreaks have been reported in Nepal as of June 2025, including 47 outbreaks in domestic pigs and one in a wild boar. The outbreaks have been distributed across the years as shown in Table 1.

**Table 1:** Distribution of ASF outbreaks in Nepal from March 2022 to June 2025.

Year	Domestic pigs	Wild boar	Total outbreaks
2022	33	0	33
2023	6	1	7
2024	5	0	5
2025 (until June)	3	0	3

Note: Data sourced from WOAH-WAHIS Animal Disease Events Management [31]; https://wahis.woah.org/#/event-management. Abbreviations: ASF, African swine fever; WOAH, World Organisation for Animal Health; WAHIS, World Animal Health Information System.

Although the number of reported outbreaks has declined in 2023–2025, isolated cases continue to emerge, underscoring the ongoing challenge of fully containing the disease. The only reported case of ASF in wild boar in Nepal was documented on 28 February 2023, in Suklaphanta National Park, Sudurpaschim Province [32]. This marks the first confirmed wildlife case in Nepal, highlighting the need for enhanced surveillance in wildlife populations to monitor potential spillover or further outbreaks. While no further cases in wild boar have been documented, the potential for future outbreaks in this and other national parks across Nepal remains a significant concern.

### 4.1 Spatiotemporal distribution of ASF in Nepal

#### 4.1.1 Spatial distribution patterns

**Fig. 4:**
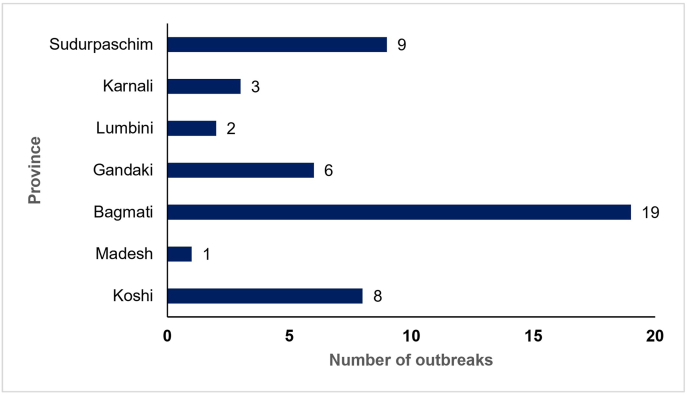
Total number of ASF outbreaks reported by province in Nepal between March 2022 and June 2025 (data sourced from WOAH-WAHIS Animal Disease Events Management [31]; https://wahis.woah.org/#/event-management). Abbreviations: ASF, African swine fever; WOAH, World Organisation for Animal Health; WAHIS, World Animal Health Information System.

ASF outbreaks have been reported across all seven provinces of Nepal, affecting 21 out of 77 districts. Bagmati Province reported the highest number of outbreaks (19), followed by Sudurpaschim (9) and Koshi (8). Lumbini and Madesh showed the lowest number of outbreaks, with two and one respectively (Fig. 4) [31]. This pattern shows clusters concentrated in the central, eastern, and far-western provinces, with relatively fewer outbreaks in the southern and mid-western provinces, indicating significant regional variations in the distribution of the outbreaks.

**Fig. 5:**
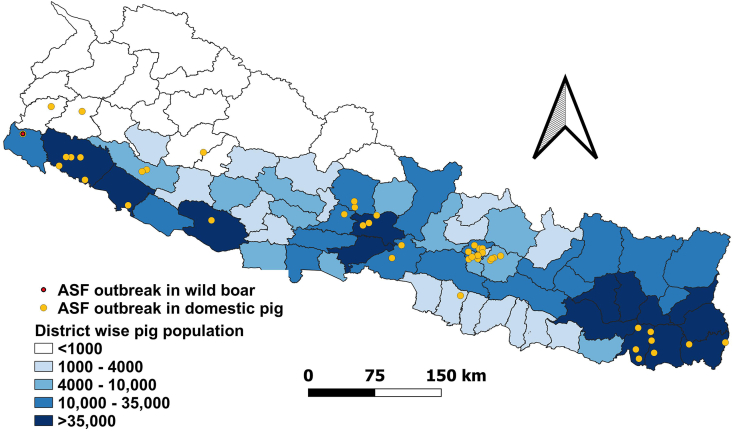
Distribution of ASF outbreaks in domestic pigs and wild boar between March 2022 and June 2025 overlaid on district-level pig population density (in thousands) across Nepal, shown by color gradient from lowest to highest density (data sourced from Statistical Information on Nepalese Agriculture 2022/23 and WOAH-WAHIS Animal Disease Events Management [26,31]; https://giwmscdnone.gov.np/media/pdf_upload/MOALD-Statical-Book-Magre-2081-Final_wgfs8ph.pdf, https://wahis.woah.org/#/event-management; map created by the authors using QGIS software, version 3.22). Abbreviations: ASF, African swine fever; WOAH, World Organisation for Animal Health; WAHIS, World Animal Health Information System.

Mapping the district-wise distribution of pig populations alongside outbreak locations suggests a potential association between ASF occurrence and pig density (Fig. 5) [26,31]. Districts with high pig densities, such as Morang (158,169 pigs), Jhapa (112,050 pigs), and Sunsari (70,699) in Koshi Province, reported multiple outbreaks. Similarly, Bara (3898 pigs), the district with the highest pig population in Madesh Province, reported the only outbreak in the region. In Sudurpashchim Province, Kailali (64,060 pigs) and Kanchanpur (18,039 pigs), which have the highest pig populations, reported five and two outbreaks, respectively. A comparable pattern was observed in Lumbini, Karnali, and Gandaki provinces, where outbreaks were recorded in districts with relatively higher pig populations [26,31]. Statistical analysis revealed a weak positive correlation between pig population size and outbreak frequency (Spearman’s *ρ* = 0.30, *P* = 0.006). While this correlation is statistically significant, its modest strength suggests that only a small proportion of the variation in outbreak frequency is explained by pig population size alone. As a result, although there is a slight tendency for more outbreaks in districts with larger herds, this relationship is not deterministic, and other risk factors are likely crucial in influencing the observed spatial distribution.

However, this trend was not consistent across all districts. Notably in Bagmati Province, Kathmandu (11,710 pigs) and Lalitpur (9458 pigs), despite having moderate pig populations compared to Chitwan (24,258 pigs), reported more outbreaks (five each) than Chitwan (three outbreaks). Additionally, some districts with low pig populations, such as Jajarkot (500 pigs) in Karnali Province and Dadeldhura (832 pigs) in Sudurpaschim Province, reported ASF outbreaks, while certain high-density districts remained unaffected [26,31]. These spatial patterns suggest that while pig population density may influence ASF dynamics, other factors, primarily movement patterns and trade dynamics, contribute to outbreak occurrence and spread [33]. In areas like Kathmandu and Lalitpur, frequent animal movement, intensive trade networks, and human-mediated transmission through transportation hubs and centralized meat processing and distribution systems may have facilitated virus introduction. In contrast, in remote districts like Jajarkot and Dadeldhura, outbreaks may be attributed to informal pig trade, limited veterinary services, low awareness, and poor biosecurity, allowing virus transmission even in small pig populations.

In addition to outbreak distribution, the burden of ASF varied across provinces, with significant differences in the number of susceptible, infected, and deceased pigs. Koshi and Bagmati provinces exhibited the highest disease burden, with Koshi reporting the most susceptible pigs and cases, as well as the highest mortality. In contrast, Madesh had the lowest numbers for all categories. Provinces like Gandaki, Lumbini, Karnali, and Sudurpaschim showed moderate levels of ASF cases and mortality. A detailed breakdown of the data, including the numbers of susceptible pigs, reported cases, and deaths across provinces, is presented in Table 2. The overall case fatality rate (CFR) was 92.91 %, based on 18,308 reported cases and 17,005 deaths out of 27,090 susceptible pigs. These CFR values, often exceeding 90 %, are consistent with outbreaks caused by highly virulent strains and reflect limited capacity for early detection and intervention.

**Table 2:** ASF outbreak data by province and pig type (domestic/wild) in Nepal, March 2022–June 2025.

Province	Pig type	Susceptible	Case	Death	CFR (%)
Koshi	Domestic	12,826	8549	8454	98.88
Madesh	Domestic	77	36	36	100.00
Bagmati	Domestic	10,353	8158	7059	86.53
Gandaki	Domestic	696	560	514	91.79
Lumbini	Domestic	720	393	352	89.57
Karnali	Domestic	254	168	155	92.26
Sudurpaschim	Domestic	2164	444	435	97.97
	Wild	1500	1	1	100.00
Total	Domestic + Wild	27,090	18,308	17,005	92.91

Note: Data sourced from WOAH-WAHIS Animal Disease Events Management [31]; https://wahis.woah.org/#/event-management. Abbreviation: ASF, African swine fever; WOAH, World Organisation for Animal Health; WAHIS, World Animal Health Information System; CFR, case fatality rate.

#### 4.1.2 Temporal distribution

**Fig. 6:**
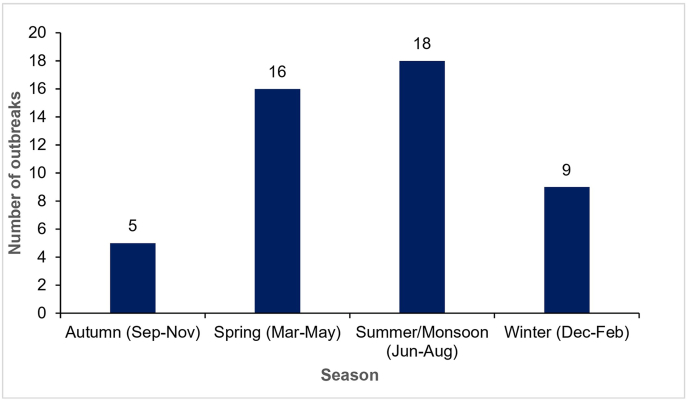
Seasonal patterns of ASF outbreaks in Nepal between March 2022 and June 2025 (based on outbreak start dates) (data sourced from WOAH-WAHIS Animal Disease Events Management [31]; https://wahis.woah.org/#/event-management). Abbreviation: ASF, African swine fever; WOAH, World Organisation for Animal Health; WAHIS, World Animal Health Information System.

The seasons and months of the ASF outbreaks were determined based on the outbreak start dates as reported in the publicly available WAHIS Animal Disease Events Management database [31]. The temporal distribution of ASF outbreaks in Nepal from March 2022 to May 2025 reveals distinct seasonal patterns, based on climatic factors such as temperature and rainfall, which vary across Nepal’s diverse eco-climatic zones. The highest number of outbreaks occurred during the summer/monsoon season (June–August) (18), characterized by high humidity and rainfall, followed by spring (March–May) (16), characterized by mild temperatures and moderate rainfall. Fewer outbreaks were recorded during winter (December–February) (9), which is cold and dry, and autumn (September–November) (5), characterized by cooler temperatures and the transition from the monsoon (Fig. 6).

The chi-square test for independence (*χ*^2^ = 9.17, *P* = 0.027) revealed a statistically significant association between season and outbreak frequency. This suggests that ASF outbreaks are not uniformly distributed throughout the seasons, indicating that certain seasons may be associated with a higher incidence of outbreaks. The seasonal variation may be associated with environmental factors such as altitude, seasonal humidity, and temperature variation [34]. Additionally, seasonal increases in pork demand, driven by cultural events and festivals, may lead to increased trade and movement of pigs, further contributing to the spread of ASF during these peak periods.

**Fig. 7:**
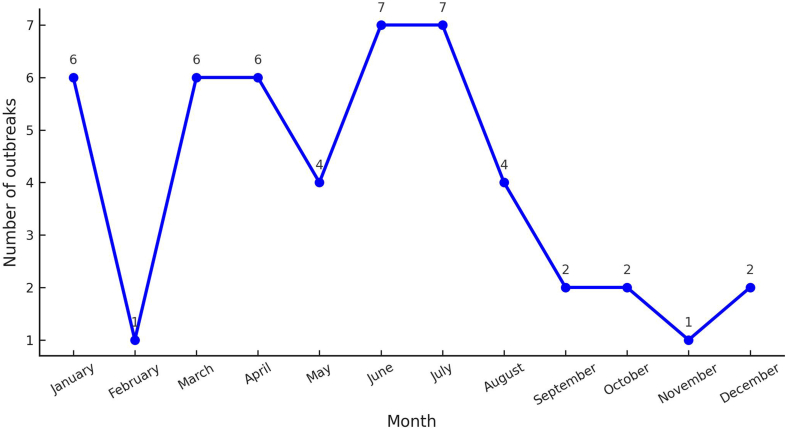
Monthly patterns of ASF outbreaks in Nepal between March 2022 and June 2025 (based on outbreak start dates) (data sourced from WOAH-WAHIS Animal Disease Events Management [31]; https://wahis.woah.org/#/event-management). Abbreviation: ASF, African swine fever; WOAH, World Organisation for Animal Health; WAHIS, World Animal Health Information System.

On a monthly level, however, the chi-square test for independence (*χ*^2^ = 15.00, *P* = 0.182) did not show a statistically significant difference, indicating that ASF outbreaks are relatively evenly distributed throughout the months. This suggests that while seasonality plays a significant role, month-to-month variability may be influenced by sporadic events such as surge in pig movement, trade, or local biosecurity breaches. Notably, the highest number of outbreaks occurred in June and July, with seven outbreaks each, coinciding with the peak of the monsoon season. This could be linked to wet weather, which increases ASF virus survival in the environment and facilitates transmission. Additionally, the monsoon season’s increased humidity and flooding may compromise biosecurity measures, reduce proper sanitation, and increase reliance on contaminated water resources, all of which facilitate virus transmission. On the other hand, outbreaks were least frequent in February and October, each recording only one outbreak (Fig. 7), possibly due to lower humidity and the absence of seasonal trade movements or pig transportation associated with cultural festivities.

### 4.2 Risk factors associated with ASF outbreaks in Nepal

The occurrence and spread of ASF in Nepal are driven by multiple, interconnected risk factors shaped by the country’s socio-economic conditions, pig farming practices, environmental interactions, and cross-border movements. Human-driven practices, including swill feeding, improper butchering of infected pigs, and the unregulated movement of pigs, significantly facilitate ASF transmission and its persistence in both local environments and wildlife populations. Although ASF is not a zoonotic disease, these anthropogenic activities play a crucial role in the dissemination and persistence of the ASF virus due to contamination of the environment. Understanding these factors is essential to address the ongoing challenges in controlling ASF outbreaks. Below are the potential major risk factors contributing to ASF outbreaks in Nepal.

#### 4.2.1 Swill feeding and contaminated feeds

Swill feeding, the practice of feeding pigs with food waste and kitchen scraps, poses a significant risk for the introduction and spread of ASF [35]. Improperly cooked or uncooked swill containing ASF-contaminated pork products can act as a direct source of viral transmission [36]. In Nepal, this practice is common among smallholder pig farmers, who frequently collect leftover food from restaurants, hotels, and canteens as a primary feed source. Due to the low economic status of many Nepalese pig farmers, swill feeding is a popular and cost-saving practice, but it is often carried out without proper processing or cooking [37,38]. The widespread reliance on swill feeding, often conducted without adequate heat treatment to inactivate the virus, significantly increases the risk of ASF introduction and spread [35]. Furthermore, the presence and movement of scavenging pigs, which have access to untreated waste and scraps, acts as a significant risk factor, further facilitating the persistence and spread of the virus. Swill feeding bans are rarely implementable or enforceable in Nepal, as it is often the only affordable way to keep pigs in low-resource settings. However, to mitigate the risk, practical measures, such as boiling leftover food at 70 °C for 30 min [39], or feeding safe, locally available alternatives like spent fruit and vegetables, food factory non-meat rejects such as stale bakery goods and broken noodles, and agricultural by- and waste products, could help reduce ASF transmission while considering the resource constraints of farmers.

Beyond swill, contaminated commercial feeds or feed ingredients represent another potential source of ASF outbreaks. Contamination can occur during manufacturing, storage, or transportation, especially if biosecurity measures are inadequate [40]. The involvement of complex global trade networks in feed production and distribution suggests a credible risk of ASF virus introduction via this pathway [41]. While there have been no confirmed cases of ASFV contamination in feed ingredients in Nepal to date, incidents have been reported in other regions. For example, in 2018, contaminated pig feed caused at least two independent ASFV introductions in China, with the virus detected in dried blood pig feeds [42]. In Nepal, the sharing of leftover feed between pig farmers is a common practice, which may further contribute to the potential spread of ASF.

#### 4.2.2 Inadequate biosecurity measures

The lack of adequate biosecurity measures on farms is one of the major contributors to the spread of ASF [43]. This risk is particularly high in Nepal, where smallholder pig farms dominate the swine sector. These farms often operate with minimal resources and limited awareness of biosecurity practices, making them highly vulnerable to ASF introduction and transmission. New animals are commonly introduced into the farms without quarantine or health checks, and there are typically no restrictions on visitors, increasing the risk of viral entry [44]. Poor hygiene, such as failure to disinfect equipment or change clothing, further facilitates transmission. Improper disposal of carcasses, such as discarding them in rivers or on roads, further increases the risk of disease transmission, complicating biosecurity efforts [45]. In Nepal, where pig farming is commonly practiced around household premises, the close proximity between pig shelters and human dwellings increases the risk of virus spread through frequent human–pig interactions.

#### 4.2.3 Cross-border movement and illegal trade

Nepal’s porous borders with India and China facilitate the illegal movement of pigs and pig products, significantly increasing the risk of introducing ASF into the country [38]. The live pig trade across borders and within the country presents a major risk, as infected animals or contaminated materials can easily be transported, introducing the virus into new areas [46]. This is evident from the districts bordering India, such as Jhapa (2 outbreaks), Sunsari (4 outbreaks), Morang (2 outbreaks), Kailali (5 outbreaks), and Kanchanpur (2 outbreaks), all of which have recorded multiple ASF outbreaks. The movement and marketing of pigs from these infected areas is a key human-driven factor in the spread of ASF. This issue is particularly pronounced due to weak border controls, inadequate veterinary inspections, and the presence of informal slaughtering and distribution networks [37]. Additionally, the smuggling of pork products, often processed or frozen, poses a hidden risk, as ASF can survive for extended periods in contaminated meat [40]. Together, these factors create a high-risk environment for ASF outbreaks, enabling rapid spread through informal trade networks, unregulated pig and pork product movements, and a lack of proper traceability in the supply chain.

#### 4.2.4 Interactions between domestic pigs and wild boars

Wild boars, which are widely distributed across Nepal’s vast forested landscapes, often overlap with domestic pig populations, potentially resulting in spillover events [32]. While comprehensive data on wild boar populations and distribution across Nepal remains limited, the Indian crested boar (*Sus scrofa cristatus*), is recognized as a widely distributed subspecies in Nepal [47]. The case of ASF in a wild boar in Shuklaphanta National Park in the Far-Western Region of Nepal marked the first such case in the country, indicating a likely spillover from domestic pigs and highlighting the vulnerability of wildlife to ASF [32]. In Nepal, where interactions between wildlife and human activities are frequent, particularly in regions where agriculture and livestock farming are prevalent, the situation becomes even more vulnerable to ASF [48]. Overlapping habitats and shared resources, such as water sources and feed, increase the risk of virus transmission, as infected domestic pigs or wild boars can contaminate these resources, leading to subsequent outbreaks [49]. These risks are especially pronounced in outdoor-rearing systems where domestic pigs are allowed to roam freely, increasing the likelihood of direct or indirect contact with wild boars.

The risk of ASF introduction in Nepal through wild boars also remains a significant concern, particularly due to the lack of physical barriers between India and Nepal’s national parks and conservation areas, which allows wild Suidae to enter the country. The role of wild boars in the transmission and spread of ASF, including the introduction of new variants, is well-documented in Europe [50] and Asia [51]. Major outbreaks have occurred in Asian countries such as China, Myanmar, Vietnam, Republic of Korea, Democratic People’s Republic of Korea and Thailand, where wild boar movement across borders has facilitated the spread of ASF[51,19] [19,51]. This highlights the critical need for regional coordination and increased surveillance to prevent the spread of ASF, particularly in areas with significant wild boar populations.

#### 4.2.5 Poor access to veterinary services

The lack of accessible veterinary services and limited disease awareness among farmers increase the risk of undetected ASF outbreaks, as cases may go unreported or misdiagnosed [52]. In Nepal, this challenge is particularly evident in remote areas, where farmers often rely on informal sources for animal health advice. Many farmers, particularly those operating smallholder and backyard farms, lack the knowledge and experience needed to manage infectious and zoonotic diseases of livestock, including ASF [53,54]. Instead of consulting trained veterinarians, they commonly turn to agro-vet shop staff, who may not have the expertise to recognize or control ASF. This reliance on informal care leads to a lack of reliable information about how ASF spreads and the measures that can be taken to prevent it, significantly heightening the risk of outbreaks and hampering effective disease control.

#### 4.2.6 Social-cultural practices

Customary communal pig slaughter, particularly during religious and traditional festivals, remains prevalent in several ethnic groups, where pork serves as a primary meat source. These slaughters often occur under unhygienic conditions, and the meat is widely shared within communities, unintentionally facilitating the spread of the virus [55]. The unregulated transport of pigs during festive and cultural events further increases the risk of spreading ASF across regions. A critical anthropogenic factor is the butchering of diseased pigs and the distribution of the meat with bone to relatives and neighbors, who eat the meat and dispose of the bones outside, where scavenging pigs are frequent visitors. Such practices significantly accelerate the spread of ASF and contribute to contamination and persistence of the ASF virus in the environment. This issue is further exacerbated in economically disadvantaged communities, where the consumption or sharing of meat from animals that have died from undiagnosed causes is common [44]. In addition, farmers may carry out emergency sales of sick pigs to mitigate economic loss. These socio-economic and cultural practices significantly increase the risk of ASF transmission, especially when infected meat enters local food chains or is shared among households during periods of increased animal movement. This poses serious challenges to disease containment, particularly in under-resourced rural settings where veterinary oversight is minimal [56].

#### 4.2.7 High pig density and seasonal variations

Factors such as seasonal temperature fluctuations (summer, autumn, spring, and winter), associated humidity levels, and altitude [34], along with high pig population and farm densities [57], have been shown to significantly increase the risk of ASF transmission. Our analysis of the published epidemiological data from recent outbreaks also suggests that these factors, particularly seasonal variation and pig population density, may play a key role in influencing ASF transmission dynamics in Nepal [31]. Seasonal analysis of outbreak data indicates that ASF incidence peaks during the summer and monsoon seasons (June–August), with fewer cases occurring in autumn and winter. Furthermore, districts with higher pig populations, including Morang, Jhapa, Sunsari, Chitwan, and Kailali, have reported multiple outbreaks, supporting the notion that pig density could be a significant key risk factor for ASF spread. High pig density may also correlate with increased trade, more frequent live animal transport, and lower per-capita biosecurity, all of which can contribute to the increased risk of ASF transmission. Additionally, cultural events and festivals such as Maghi (celebrated by the Tharu in the Terai and the Magar in the western and central hills), Udhauli (celebrated by the Rai and Limbu communities), and Lhosar (celebrated by the Tamang community), which are predominantly observed in the eastern regions of Nepal, often lead to a seasonal surge in pork demand and pig movement, further exacerbating the risk of ASF transmission during peak periods.

Together, these spatial and temporal trends emphasize the importance of incorporating both pig density and seasonal variation into ASF risk assessments. This could inform targeted control measures such as seasonal surveillance intensification, zoning based on pig density, and movement restrictions during high-risk periods to mitigate ASF transmission effectively.

### 4.3 Impacts of ASF in Nepal

**Fig. 8:**
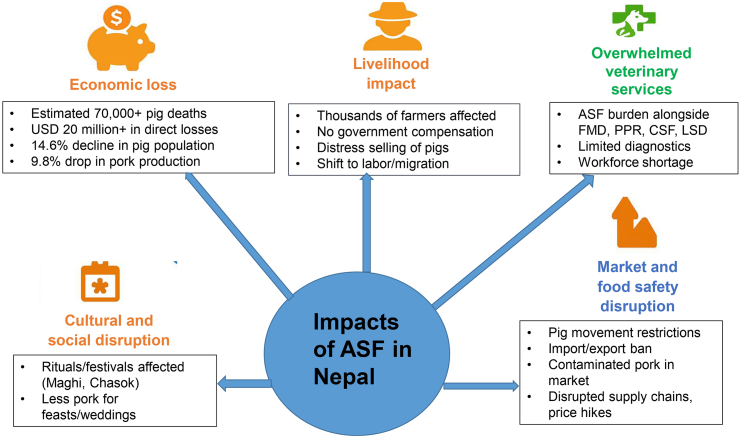
Multidimensional impacts of ASF outbreaks in Nepal (March 2022–June 2025). Abbreviations: ASF, African swine fever; FMD, foot and mouth disease; PPR, peste des petits ruminants; CSF, classical swine fever; LSD, lumpy skin disease.

ASF outbreaks in Nepal have had a profound impact on the country’s pig farming sector (Fig. 8). The economic consequences have been particularly severe, with significant financial losses due to high pig mortality and disruptions in the pork market. Although the officially reported pig mortality due to ASF stood at 17,005 by June 2025 [31], the actual impact appears significantly worse due to widespread underreporting. This underreporting is likely driven by factors such as the lack of compensation for affected farmers, fear of stigma, and limited veterinary surveillance, which hinder effective disease control and response efforts. Inaccurate mortality reporting impedes the timely identification of outbreak hotspots, delays response measures, and undermines the allocation of resources for containment. While official figures are limited, estimates based on anecdotal field reports and non-official sources suggest approximately 70,000 pigs may have died from ASF in Nepal [58]. This equates to an estimated direct economic loss of 20 million US dollars, based solely on the market value of the pigs lost. This represents a relatively huge amount for smallholder farmers, who were primarily affected. However, the overall economic impact is likely to be significantly greater when accounting for indirect losses. These includes the loss of valuable breeding stock, which hinders future production; reduced restocking rates due to fear of ASF reoccurrence; the costs associated with upgrading biosecurity measures; and a decline in the market value of pigs. While quantifying these losses is challenging, their cumulative effect is expected to be substantial. Between 2021/22 and 2022/23, Nepal experienced a 14.5 % reduction (231,000 pigs) in its total swine population and a 9.8 % decline (3526 MT) in pork production [26], likely influenced by ASF outbreaks and their broader impact on pig farming in Nepal. This has further impacted the economic contribution of the pig sector, which, prior to the ASF outbreak, had an annual demand growth of approximately 10 % and contributed significantly to Nepal’s meat production [26].

Besides direct economic losses, ASF has severely affected the livelihoods of farmers who rely primarily on pig farming as their main source of income. In Nepal, approximately 422,501 families are engaged in pig farming [59], many of whom belong to marginalized, rural, or indigenous communities with limited economic opportunities. The sudden death of pigs, fluctuations in pork prices, and lack of government support have left these families struggling financially. Consequently, many pig farmers are either shifting to other forms of livestock farming, seeking daily wage labor, or migrating abroad in search of better employment opportunities. These shifts in livelihood, including labor migration, may have contributed to a reduction in local pork production capacity in Nepal. While the exact extent of this impact remains unclear due to the lack of specific data, this is an area that warrants further analysis to better understand its implications for the pig farming sector. Additionally, in many affected areas, it is reported that farmers sell sick pigs at low prices to butcheries in an attempt to minimize financial losses before the animals die, potentially from ASF. These reports, though anecdotal, are recurring in many affected regions. While ASF is not a zoonotic disease, this practice raises concerns about food safety and the potential risks associated with the consumption of poorly handled or infected meat, which could affect meat quality and lead to contamination. ASF has also disrupted the pork supply chain due to restrictions imposed on both internal and international trade. Following the outbreak, Department of Livestock Services (DLS), in coordination with District Administration Offices, issued directives to strictly control the movement of pigs and pig products to and from affected areas within the country. Quarantine measures and mandatory health certification have been enforced, disrupting traditional trade routes and systems, severely impacting local farmers and businesses. However, enforcement remains uneven and under-resourced, with limited active surveillance of pig movements. This has led to a decline in pork supply, driving up prices and threatening food security, particularly for rural communities and ethnic groups for whom pork is an affordable and key dietary protein source. Furthermore, international trade has been affected, with countries such as the United States imposing bans on Nepali pork products due to ongoing ASF outbreaks [60]. While Nepal’s pork exports constitute a small fraction of the country’s overall agricultural trade, the U.S. ban, though perhaps symbolic, has nonetheless affected the credibility of Nepal’s pork trade. Nepal had proactively enforced a ban on the importation of pigs and pig-derived products from countries affected by ASF, as formally announced in the Nepal Rajpatra (official gazette) in January 2019 [37].

ASF has also significantly disrupted the socio-cultural practices of indigenous communities in Nepal, where pig farming is deeply tied to both livelihood and cultural rituals. For many ethnic groups, such as the Rai, Limbu, Tharu, and Magar, pigs play a central role in religious ceremonies and traditional festivals. The outbreaks of ASF have interrupted these cultural practices, resulting in a broader loss of cultural continuity and ritual cohesion. The inability to carry out traditional animal sacrifices and prepare pork-based dishes for communal gatherings and major celebrations has affected the cultural fabric of these communities, limiting participation in important rites and weakening social ties [24,61,62]. In addition, the ASF outbreak has placed significant strain on Nepal’s veterinary services. The sector is already burdened with managing recent outbreaks of lumpy skin disease (LSD, 2020) [63] and Glanders (2021) [64], alongside avian influenza (recurring since 2009) [65], and endemic diseases such as porcine reproductive and respiratory syndrome (PRRS) [66], foot and mouth disease (FMD) [67], peste des petits ruminants (PPR) [68], Rabies [69,70], and classical swine fever (CSF) [71]. The cumulative burden of multiple transboundary and endemic diseases, including ASF, has overwhelmed Nepal’s limited veterinary infrastructure, particularly in remote areas, limiting the capacity for effective disease surveillance and response.

## 5 Discussion

This section discusses the major challenges and critical gaps in controlling ASF in Nepal and offers future recommendations to enhance disease prevention and control efforts.

### 5.1 Control challenges of ASF in Nepal

**Fig. 9:**
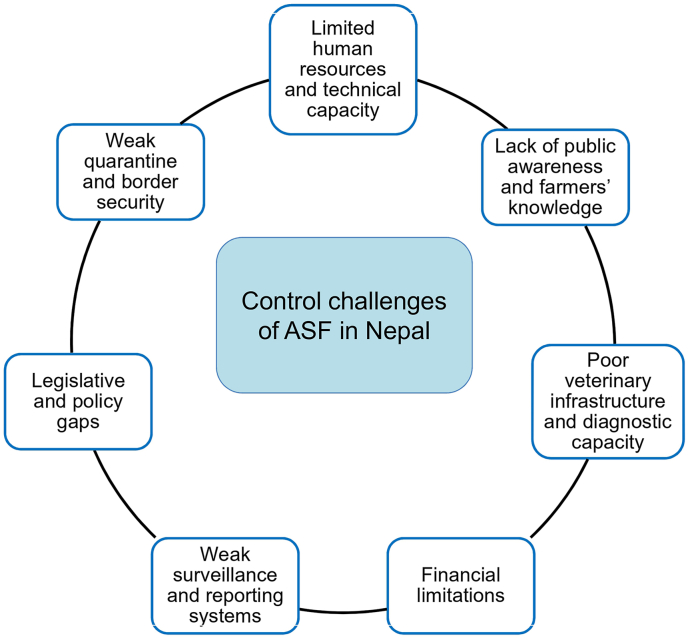
Major challenges hindering ASF control in Nepal. Abbreviation: ASF, African swine fever.

ASF has been present in Nepal for over three years, severely impacting the pig farming sector. Despite efforts by the DLS, such as restricting the domestic movement of pigs and pig products and urging farmers to adhere to biosecurity measures, these actions have failed to contain the spread of ASF [72]. Key control measures, including active surveillance, proper disposal of carcasses, culling or isolation of infected pigs, and quarantining suspected animals, have been inadequately enforced, further facilitating the spread. Several challenges have hindered the effective implementation of ASF control measures in Nepal (Fig. 9).

A major challenge in controlling ASF is the severe shortage of veterinary personnel, particularly at the local level. Many municipalities lack field veterinary staff, and the emigration of veterinarians has exacerbated the problem [73]. This shortage is compounded by inadequate training and a lack of professional development opportunities, which weakens the capacity of existing personnel to handle ASF outbreaks effectively. The insufficient veterinary infrastructure, especially in remote and mountainous areas, also hinders timely disease detection and response. The CVL in Kathmandu, the only facility capable of performing molecular detection of ASF, is overwhelmed due to limited resources and high sample volumes, causing delays in confirming cases. Provincial veterinary laboratories rely on less sensitive rapid antigen tests, which are not suitable for large-scale ASF diagnosis.

Financial constraints are another significant barrier to effective ASF control. While the DLS budget has seen slight increases, it remains insufficient to support long-term disease control programs [73]. There is no rapid mobilization mechanism for emergency funds, which delays responses during outbreaks. Local governments face similar financial limitations, with allocations primarily for basic animal health services. The absence of a compensation scheme for ASF-related losses, unlike in the case of avian influenza, further limits resources available for response [74]. Additionally, weak surveillance systems, particularly for active monitoring, and the lack of an animal identification and traceability system hamper the ability to detect and control outbreaks in a timely manner [75]. Reporting is often inconsistent, and the DLS lacks the legal authority to enforce mandatory disease reporting from local governments and private stakeholders, which undermines the effectiveness of surveillance [76]. Legislative and policy gaps further hinder effective ASF control. Existing frameworks, such as the Animal Health and Livestock Services Act (1999), do not specifically address the prevention and control of transboundary animal diseases like ASF. The Slaughterhouse and Meat Inspection Act (1998) remains unimplemented, and the absence of a clear enforcement strategy means meat often enters the market without proper inspection [77]. The division of responsibilities among federal, provincial, and local governments, coupled with the restructuring of Nepal into a federal system, has fragmented the veterinary service delivery, causing delays and coordination issues. [75,76]. These institutional weaknesses significantly compromise Nepal’s ability to implement disease control measures.

Nepal’s weak quarantine infrastructure and porous border with India also contribute to the challenge [37,38]. The country’s quarantine facilities are insufficient, with limited staff and inadequate infrastructure, allowing the unregulated movement of pigs and pig products. This situation is exacerbated by the lack of formal coordination with neighboring countries like India and China, making it difficult to prevent the spread of ASF and other transboundary animal diseases [78]. Compounding these challenges, public awareness of ASF remains low, with many smallholder pig farmers lacking accurate information on how the disease spreads and how to prevent it [38]. The absence of national communication campaigns and limited farmer training on biosecurity practices has led to inconsistent implementation of disease control measures at the farm level. This lack of awareness delays reporting and hampers community-level efforts to control ASF.

These challenges underscore the urgent need for a comprehensive approach to strengthen veterinary services, improve disease surveillance, enhance policy enforcement, and raise public awareness to effectively manage and control ASF in Nepal.

### 5.2 Future recommendations to prevent and control ASF in Nepal

To prevent and control ASF in Nepal, a comprehensive, multi-faceted approach is needed. The following recommendations aim to strengthen the country’s response to ASF by addressing key areas such as surveillance, biosecurity, infrastructure, and collaboration, all of which are critical for effective control and mitigation.

#### 5.2.1 Strengthening surveillance and early detection systems

A robust disease surveillance program should be established to enable early detection and timely containment of ASF outbreaks. High-risk sites, such as border quarantine stations, commercial pig farms, retail pig markets, and livestock transportation routes, should be prioritized [79]. Concurrent sample collection at these sites will aid in early detection. Enhancing the National Animal Health Information System through continuous training and infrastructure upgrades is essential [80]. Additionally, a pig value chain study should be conducted to understand ASF transmission risks and design an evidence-based surveillance system [20].

#### 5.2.2 Strengthening veterinary and diagnostic infrastructure

To strengthen veterinary and diagnostic infrastructure, it is crucial to expand diagnostic capacity at provincial and local levels to reduce reliance on central laboratories and accelerate outbreak response. Laboratories should be equipped for molecular testing, and gene-sequencing capabilities should be established at the CVL for pathogen characterization [81]. Additionally, a rapid response team with trained field personnel should be developed to ensure quick and efficient action during outbreaks [82].

#### 5.2.3 Promoting farm-level biosecurity measures

Biosecurity protocols should be rigorously implemented on farms, particularly in commercial operations. Essential practices include installing perimeter fencing, using footbaths, regulating movement, and isolating new pigs [79]. For smallholder farms with limited resources, affordable solutions like temporary barriers and designated isolation areas can be adapted [83]. It is also important to ensure compliance with Good Animal Husbandry Practices and provide targeted biosecurity training to farm personnel [20,84].

#### 5.2.4 Enhancing cross-border security and international collaboration

Strengthening controls on cross-border and internal pig movements will help prevent ASF transmission [85]. A national pig traceability system, along with mandatory documentation, will help track movements and identify potential hotspots. Regional cooperation should be promoted through the Global Framework for the Progressive Control of Transboundary Animal Diseases, prioritizing transboundary animal diseases, including ASF, and formulating and implementing appropriate strategies for its progressive control [86,87]. In the current geopolitical context, the Bay of Bengal Initiative for Multi-Sectoral Technical and Economic Cooperation may serve as a valuable platform for fostering regional collaboration.

#### 5.2.5 Raising public awareness and farmer education

Raising public awareness and educating farmers are critical to preventing ASF spread. Farmers should be educated about ASF transmission, clinical signs, and their role in preventing the disease. Community-based platforms, such as Farmers’ Field Schools and awareness campaigns, should be utilized to reach farmers, particularly women involved in basic husbandry [88,89]. Additionally, awareness of ASF’s impact on food security and livelihoods can be raised through social media, radio, and print media [90].

#### 5.2.6 Develop legal and strategic frameworks for ASF control

To strengthen ASF control, priority should be given to developing an ASF Control Regulation or integrating it into existing frameworks, such as the Animal Health and Livestock Services Act (1999). The Slaughterhouse and Meat Inspection Act (1999) should be enforced to prevent ASF transmission through contaminated pork products [77]. Additionally, the Zoning and Compartmentalization Directive (2022) should be implemented to establish disease-free zones [91]. A compensation mechanism for ASF-related losses should be introduced to encourage compliance and support recovery.

#### 5.2.7 Monitor and control domestic-wild boar interaction

Targeted monitoring in high-risk buffer zones near protected areas is essential to prevent ASF transmission between wild and domestic pigs [92]. Camera traps, community reporting systems, and surveys can be used to monitor wild boar populations and their movements [93,94]. Additionally, wild boar carcasses should be tested promptly, and fencing around high-risk pig farms should be used to further reduce transmission [95].

#### 5.2.8 Community and stakeholder engagement

Engaging local communities, farmers, and stakeholders is essential for implementing practical and culturally appropriate ASF control measures [96]. A coordinated network of government, private sector, and community actors should be established to strengthen ASF control efforts [97]. Additionally, incorporating Risk Communication and Community Engagement approaches will help encourage behavior change and foster trust among the public [98].

## 6 Conclusion

The emergence and spread of ASF in Nepal have exposed critical vulnerabilities in the country’s animal health systems, agricultural practices, and socioeconomic structures. Beyond its devastating effects on pig populations and livelihoods, ASF has disrupted food security and cultural practices. This study underscores the interconnectedness of human, animal, and environmental health, emphasizing the urgent need for concrete, cross-sectoral measures to control ASF within the One Health framework. Strengthening veterinary infrastructure, enhancing diagnostics, promoting biosecurity, and fostering cross-border collaboration are essential priorities. Public awareness campaigns, multi-level coordination, and local capacity building are also crucial for effective disease management. Nepal’s experience with ASF offers valuable lessons for resource-limited countries, highlighting the importance of integrated risk communication, surveillance, and preparedness. Embedding ASF control within a One Health framework will enhance Nepal’s resilience to future transboundary animal diseases. It calls for inclusive governance, regional cooperation, and sustained investment in systems that recognize the shared health of animals, people, and ecosystems.

## CRediT authorship contribution statement

**Sameer Thakur:** Writing – review & editing, Writing – original draft, Visualization, Supervision, Formal analysis, Data curation, Conceptualization. **Kshitiz Shrestha:** Writing – review & editing, Writing – original draft, Data curation. **Ram Chandra Acharya:** Writing – review & editing, Writing – original draft. **Parikshya Gurung:** Writing – review & editing, Writing – original draft. **Surendra Karki:** Writing – review & editing, Writing – original draft, Supervision.

## Ethics Statement

Not applicable.

## Data availability statement

All data on ASF outbreaks in Nepal used in this review were obtained from the publicly available World Animal Health Information System (WAHIS) Event Management database, accessible at: https://wahis.woah.org/#/event-management.

## Funding

The authors did not receive any funding for the preparation and writing of this manuscript.

## Declaration of interests

The authors declare that they have no known competing financial interests or personal relationships that could have appeared to influence the work reported in this paper.
